# Crystal structures of two isotypic lanthanide(III) complexes: tri­aqua­[2,6-di­acetyl­pyridine bis­(benzoyl­hydrazone)]methano­llanthanide(III) trichloride methanol disolvates (*Ln* ^III^ = Tb and Dy)

**DOI:** 10.1107/S2056989018004103

**Published:** 2018-03-16

**Authors:** Chihiro Kachi-Terajima, Norihisa Kimura

**Affiliations:** a Department of Chemistry, Faculty of Science, Toho University, Miyama, Funabashi, Chiba 274-8510, Japan

**Keywords:** crystal structure, lanthanide(III), 2,6-di­acetyl­pyridine bis­(benzoyl­hydrazone) ligand, terbium(II), dysprosium (III), hydrogen bonding, π–π stacking, supra­molecular framework

## Abstract

The title lanthanide complexes, [*Ln*(DAPBH_2_)(CH_3_OH)(H_2_O)_3_]Cl_3_·2CH_3_OH [*Ln* ^III^ = Tb and Dy; DAPBH_2_ = 2,6-di­acetyl­pyridine bis­(benzoyl­hydrazone), C_23_H_21_N_5_O_2_], are isotypic. The central lanthanide ions are nine-coordinate, being ligated by three N and two O atoms from the penta­dentate DAPBH_2_ ligand, and four O atoms from the coordinated methanol mol­ecule and three coordinated water mol­ecules. The coordination geometry of the lanthanide ion is a distorted capped square anti­prism. In the crystals, the various components are linked by O—H⋯Cl, N—H⋯Cl and O—H⋯O hydrogen bonds, forming three-dimensional supra­molecular frameworks. Within the frameworks, there are C—H⋯Cl and C—H⋯O hydrogen bonds and offset π–π inter­actions (inter­centroid distance *ca* 3.81 Å).

## Abstract 2

Two isotypic complexes of Tb^III^ and Dy^III^ with the ligand 2,6-di­acetyl­pyridine bis­(benzoyl­hydrazone) have been synthesized and structurally characterized.

## Chemical context   

Mol­ecule-based magnets based on lanthanide ions have attracted much attention because of their large magnetic moments and magnetic anisotropy. The design of building units, such as the coordination–acceptor or coordination–donor magnetic units, is a key process in the construction of multi-dimensional magnetic materials. Some lanthanide complexes with 2,6-di­acetyl­pyridine bis­(benzoyl­hydrazone as ligand (DAPBH_2_) have been reported, *viz*. for La^III^ (Thomas *et al.*, 1979 ▸), Yb^III^ (Pan *et al.*, 1989 ▸), Eu^III^ (Gao & Wang, 2012 ▸), Dy^III^ (Batchelor *et al.*, 2014 ▸) and for La^III^ and Dy^III^ (Gao *et al.*, 2016 ▸). The Dy complexes having two DAPBH_2_ ligands (Batchelor *et al.*, 2014 ▸) have demonstrated attractive single-mol­ecule magnet behaviour, indicating that DAPBH_2_ ligands are useful for constructing magnetic units. For the use of DAPBH_2_ complexes as building blocks, coordination active sites are needed. The DAPBH_2_ ligand is penta­dentate, thus it can make coordination sites in the axial positions of the lanthanide ion. These complexes have coordinated or non-coordinated nitrate ions, which can disturb the coordination of coordination–donor units. We report herein on the Tb^III^ and Dy^III^ complexes with the DAPBH_2_ ligand containing non-coordinating chloride ions as the coordination–acceptor building units.

## Structural commentary   

**Figure 1:**
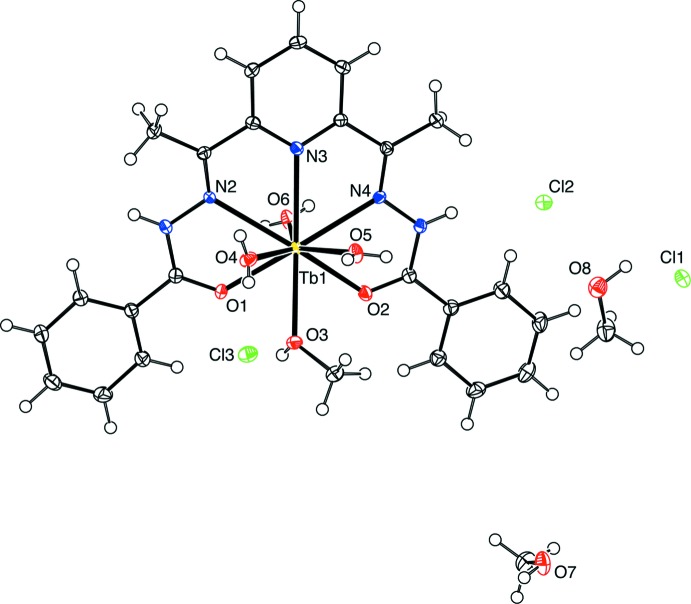
Mol­ecular structure of the Tb^III^ complex, showing the selected atom-labelling scheme. Displacement ellipsoids are drawn at the 50% probability level.

The title Tb^III^ and Dy^III^ complexes are isotypic, crystallizing in the same space group (*P* 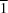) with almost identical unit-cell parameters. The representative mol­ecular structure of the Tb^III^ complex is shown in Fig. 1 ▸.
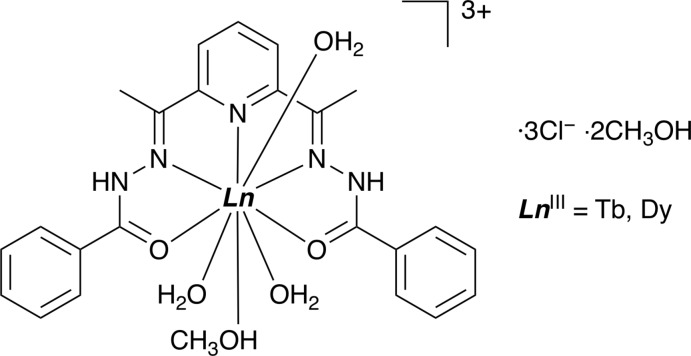


The lanthanide ion is surrounded by six oxygen atoms and three nitro­gen atoms, and the coordination polyhedron is a distorted capped square anti­prism. The equatorial coordin­ation site of the *Ln* ^III^ ion is occupied by an N_3_O_2_ atom set of a penta­dentate DAPBH_2_ ligand. Selected bond lengths and bond angles for both complexes are compared in Table 1 ▸. The *Ln*–donor bond distances are in the range of 2.321 (2)–2.596 (2) Å for the Tb^III^ complex and 2.313 (2)–2.584 (2) Å for the Dy^III^ complex. The bond distances for the Dy^III^ complex are slightly shorter than those of the Tb^III^ complex as a result of the lanthanide contraction effect. The DAPBH_2_ ligand is approximately planar, and the *Ln* ^III^ ion lies out of the mean plane (O1/N2/N3/N4/O2) by a distance of 0.5754 (3) Å for the Tb^III^ complex and 0.5702 (3) Å for the Dy^III^ complex. The coordination of the DAPBH_2_ ligand to the lanthanide ion shows a bent arrangement [bond angles O1—*Ln*—N4 and O2—*Ln*—N2 are 149.40 (6) and 152.08 (7)°, respectively, for the Tb^III^ complex, and 149.36 (7) and 151.76 (8)°, respectively, for the Dy^III^ complex]. These coordination features are similar to those reported for the dysprosium DAPBH_2_ nitrate complex (Gao *et al.*, 2016 ▸). Three water mol­ecules and one methanol mol­ecule are involved in the coordination sphere of the *Ln* ^III^ ion. The asymmetric unit consists of the *Ln* ^III^ complex, three chlorides as counter-ions, and two methanol solvent mol­ecules.

## Supra­molecular features   

**Figure 2:**
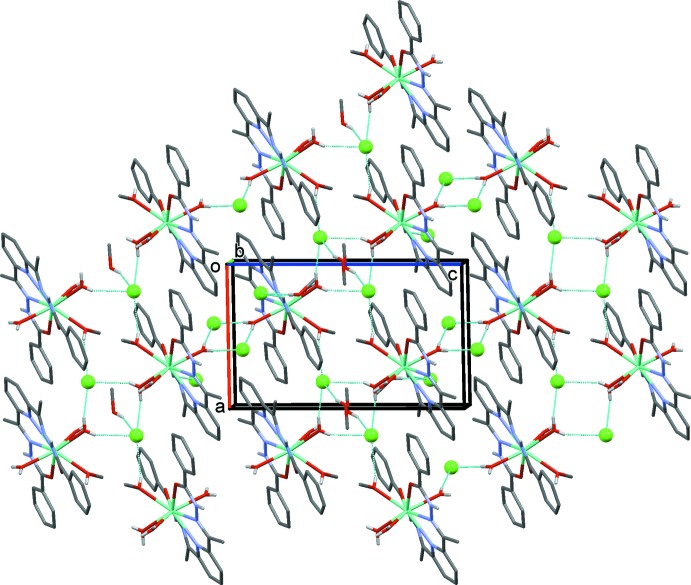
A view along the *b* axis of the hydrogen-bonded (dashed lines) layer structure of the Tb^III^ complex. The Cl^−^ ions are shown as green balls and the C-bound H atoms have been omitted for clarity.

**Figure 3:**
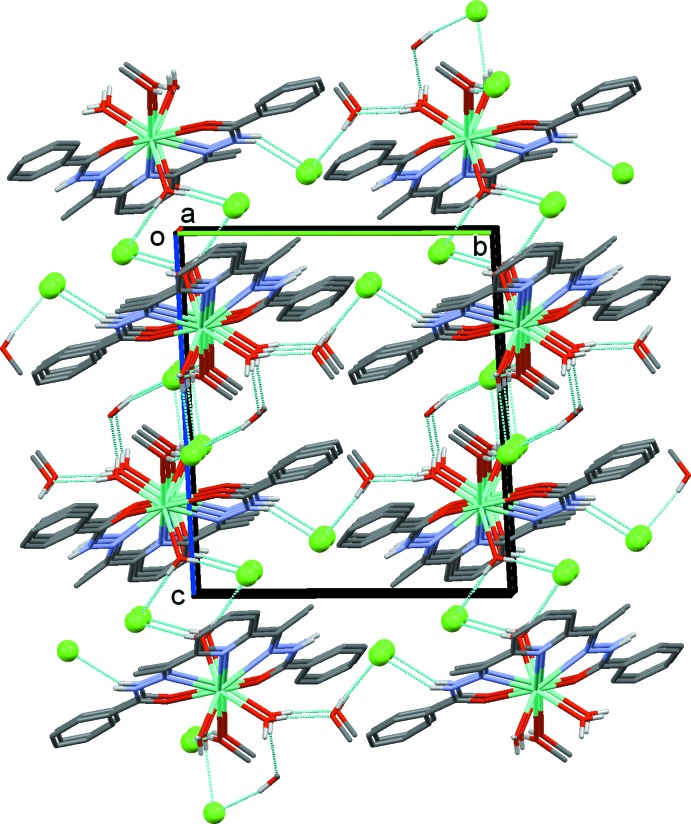
A view along the *a* axis of the hydrogen-bonded (dashed lines) supra­molecular framework of the Tb^III^ complex. The Cl^−^ ions are shown as green balls and the C-bound H atoms have been omitted for clarity.

In the crystals, the lanthanide complexes are connected by O—H⋯Cl, N—H⋯Cl, O—H⋯O, C—H⋯Cl and C—H⋯O hydrogen bonds (Tables 2 ▸ and 3 ▸). The representative crystal structure of the Tb^III^ complex is discussed here and the crystal packing is shown in Figs. 2 ▸ and 3 ▸. The various components are linked by O—H⋯Cl and N—H⋯Cl hydrogen bonds, forming layers parallel to (101), as illustrated in Fig. 2 ▸ (see also Table 2 ▸). Within the layers there are offset π–π inter­actions involving the benzoyl rings of neighbouring mol­ecules [*Cg*2⋯*Cg*3^*a*^,^*b*^ = 3.813 (2) Å, α = 3.8 (1)°, inter­planar distance = 3.483 (1) Å, slippages = 1.77 and 1.55 Å; *Cg*2 and *Cg*3 are the centroids of C2–C7 and C18–C23 rings, respectively, symmetry codes: (*a*) *x*, *y* − 1, *z*; (*b*) *x*, *y* + 1, *z*]. The layers are linked by O—H⋯O, O—H⋯Cl and N—H⋯Cl hydrogen bonds, forming a three-dimensional supra­molecular framework, which is reinforced by a series of C—H⋯Cl and C—H⋯O hydrogen bonds (Fig. 3 ▸ and Table 2 ▸).

## Database survey   

A search of the Cambridge Structural Database (Version 5.39, update February 2018; Groom *et al.*, 2016 ▸) for the DAPBH_2_ ligand gave 59 hits. There are 12 lanthanide nitrate DAPBH_2_ complexes but no complexes with halogen ions as counter-ions. A number of halides of transition metal DAPBH_2_ complexes have been reported, *viz*. Mn (Lorenzini *et al.*, 1983 ▸), Fe (Bar *et al.*, 2015 ▸), Co (Batchelor *et al.*, 2011 ▸), Cu (Neto *et al.*, 2013 ▸), and Re (Al-Shihri *et al.*, 1993 ▸).

## Synthesis and crystallization   

A methanol solution (15 ml) of TbCl_3_·6H_2_O (0.178 g, 0.48 mmol), 2,6-di­acetyl­pyridine (0.075 g, 0.45 mmol), and benzoyl­hydrazine (0.127 g, 0.93 mmol) was refluxed for 2 h. The resulting mixture was filtered. Vapour diffusion of diethyl ether into the filtrate afforded colourless plate-like crystals of the Tb^III^ complex (0.116 g, yield 30%). The synthetic procedure for the Dy^III^ complex is similar, starting from dysprosium chloride (yield 43%).

## Refinement   

Crystal data, data collection, and structure refinement details are summarized in Table 4 ▸. The O—H hydrogen atoms of the water and methanol mol­ecules were located in difference-Fourier maps and were refined isotropically. The O—H distance of the coordinated methanol mol­ecule in the Dy^III^ complex was restrained to 0.82 Å. Other hydrogen atoms were generated geometrically and refined with a riding model: N—H = 0.88 Å, C–H = 0.95–0.98 Å with *U*_iso_(H) = 1.5*U*_eq_(C-methyl, O-hydrox­yl) and 1.2 *U*_eq_(C, N) for other H atoms.

## Supplementary Material

Crystal structure: contains datablock(s) TbDAPBH2, DyDAPBH2, global. DOI: 10.1107/S2056989018004103/su5429sup1.cif

Structure factors: contains datablock(s) TbDAPBH2. DOI: 10.1107/S2056989018004103/su5429TbDAPBH2sup2.hkl

Structure factors: contains datablock(s) DyDAPBH2. DOI: 10.1107/S2056989018004103/su5429DyDAPBH2sup3.hkl

CCDC references: 1828810, 1828809

Additional supporting information:  crystallographic information; 3D view; checkCIF report

## Figures and Tables

**Table 1:** Selected geometric parameters (Å, °) for the Tb^III^ and Dy^III^ complexes

Tb1—N2	2.5845 (19)	Dy1—N2	2.577 (2)
Tb1—N3	2.596 (2)	Dy1—N3	2.584 (2)
Tb1—N4	2.5685 (19)	Dy1—N4	2.555 (2)
Tb1—O1	2.3660 (16)	Dy1—O1	2.358 (2)
Tb1—O2	2.4074 (17)	Dy1—O2	2.3961 (19)
Tb1—O3	2.4867 (18)	Dy1—O3	2.472 (2)
Tb1—O5	2.3642 (19)	Dy1—O4	2.420 (2)
Tb1—O4	2.428 (2)	Dy1—O5	2.354 (2)
Tb1—O6	2.321 (2)	Dy1—O6	2.313 (2)
			
O1—Tb1—N4	149.40 (6)	O1—Dy1—N4	149.36 (7)
O2—Tb1—N2	152.08 (7)	O2—Dy1—N2	151.76 (8)
O6—Tb1—N4	74.40 (7)	O6—Dy1—N4	74.67 (8)
O6—Tb1—O2	76.48 (7)	O6—Dy1—O2	76.31 (8)
O6—Tb1—N2	76.97 (7)	O6—Dy1—N2	76.91 (8)
O6—Tb1—N3	79.68 (7)	O6—Dy1—N3	80.29 (8)
O6—Tb1—O1	76.15 (7)	O6—Dy1—O1	75.91 (8)

**Table 2:** Hydrogen-bond geometry (Å, °) for the Tb^III^ complex[Chem scheme1]

*D*—H⋯*A*	*D*—H	H⋯*A*	*D*⋯*A*	*D*—H⋯*A*
O4—H4*SA*⋯Cl3	0.64 (4)	2.61 (4)	3.213 (2)	159 (5)
N1—H1*A*⋯Cl2^i^	0.88	2.52	3.299 (2)	148
O6—H6*SA*⋯Cl1^i^	0.73 (4)	2.32 (4)	3.040 (2)	172 (4)
O3—H3*S*⋯Cl3^ii^	0.68 (5)	2.68 (5)	3.2998 (19)	153 (5)
O4—H4*SB*⋯Cl3^iii^	0.81 (4)	2.34 (4)	3.1323 (19)	169 (4)
O6—H6*SB*⋯Cl1^iv^	0.74 (4)	2.32 (4)	3.058 (2)	176 (3)
O7—H7*S*⋯Cl2^v^	0.72 (3)	2.34 (3)	3.050 (2)	174 (4)
O8—H8*S*⋯Cl3^vi^	0.91 (4)	2.23 (4)	3.110 (2)	163 (4)
O5—H5*SA*⋯O8^vi^	0.77 (4)	1.96 (4)	2.710 (3)	166 (4)
O5—H5*SB*⋯O7^v^	0.79 (4)	1.88 (4)	2.664 (3)	168 (4)
C7—H7⋯Cl2^i^	0.95	2.74	3.491 (3)	137
C11—H11⋯Cl1^vii^	0.95	2.80	3.731 (3)	167
C12—H12⋯Cl1^viii^	0.95	2.80	3.741 (3)	172
C16—H16*B*⋯Cl2	0.98	2.66	3.628 (3)	170
C16—H16*C*⋯Cl2^viii^	0.98	2.79	3.621 (3)	143
C19—H19⋯Cl2	0.95	2.73	3.515 (3)	140
C26—H26*A*⋯Cl3^ix^	0.98	2.80	3.774 (3)	174
C4—H4⋯O8^x^	0.95	2.59	3.397 (3)	143

Symmetry codes: (i) 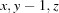; (ii) 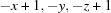; (iii) 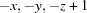; (iv) 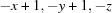; (v) 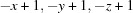; (vi) 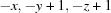; (vii) 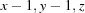; (viii) 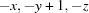; (ix) 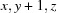; (x) 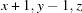.

**Table 3:** Hydrogen-bond geometry (Å, °) for the Dy^III^ complex[Chem scheme1]

*D*—H⋯*A*	*D*—H	H⋯*A*	*D*⋯*A*	*D*—H⋯*A*
O4—H4*SA*⋯Cl3	0.77 (4)	2.45 (4)	3.211 (3)	170 (4)
N1—H1*A*⋯Cl2^i^	0.88	2.53	3.298 (2)	147
O6—H6*SA*⋯Cl1^i^	0.77 (4)	2.27 (4)	3.030 (3)	168 (4)
O3—H3*S*⋯Cl3^ii^	0.79 (3)	2.60 (3)	3.329 (2)	156 (3)
O4—H4*SB*⋯Cl3^iii^	0.73 (5)	2.42 (4)	3.133 (3)	165 (4)
O6—H6*SB*⋯Cl1^iv^	0.67 (3)	2.39 (3)	3.058 (3)	179 (5)
O7—H7*S*⋯Cl2^v^	0.78 (4)	2.29 (4)	3.049 (3)	165 (4)
O8—H8*S*⋯Cl3^vi^	0.77 (5)	2.34 (5)	3.104 (3)	174 (6)
O5—H5*SA*⋯O8^vi^	0.81 (4)	1.96 (4)	2.702 (4)	154 (4)
O5—H5*SB*⋯O7^v^	0.72 (5)	1.95 (5)	2.659 (3)	167 (6)
C7—H7⋯Cl2^i^	0.95	2.75	3.494 (3)	136
C11—H11⋯Cl1^vii^	0.95	2.80	3.730 (3)	167
C12—H12⋯Cl1^viii^	0.95	2.79	3.734 (3)	172
C16—H16*B*⋯Cl2	0.98	2.66	3.620 (3)	166
C16—H16*C*⋯Cl2^viii^	0.98	2.77	3.618 (3)	145
C19—H19⋯Cl2	0.95	2.74	3.522 (3)	140
C26—H26*A*⋯Cl3^ix^	0.98	2.79	3.761 (4)	171
C4—H4⋯O8^x^	0.95	2.60	3.406 (4)	143

Symmetry codes: (i) 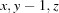; (ii) 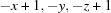; (iii) 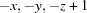; (iv) 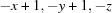; (v) 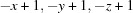; (vi) 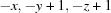; (vii) 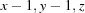; (viii) 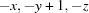; (ix) 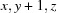; (x) 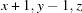.

**Table 4:** Experimental details

	Tb^III^ complex	Dy^III^ complex
Crystal data		
Chemical formula	[Tb(C_23_H_21_N_5_O_2_)(CH_4_O)(H_2_O)_3_)]Cl_3_·2CH_4_O	[Dy(C_23_H_21_N_5_O_2_)(CH_4_O)(H_2_O)_3_)]Cl_3_·2CH_4_O
*M*_r_	814.89	818.47
Crystal system, space group	Triclinic, *P* 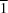	Triclinic, *P* 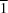
Temperature (K)	100	100
*a*, *b*, *c* (Å)	8.9703 (7), 12.6433 (9), 14.4233 (11)	8.9852 (7), 12.6242 (10), 14.3887 (12)
α, β, γ (°)	87.004 (1), 88.752 (1), 81.980 (1)	87.062 (1), 88.810 (1), 82.068 (1)
*V* (Å^3^)	1617.4 (2)	1614.2 (2)
*Z*	2	2
Radiation type	Mo *K*α	Mo *K*α
μ (mm^−1^)	2.49	2.62
Crystal size (mm)	0.20 × 0.15 × 0.05	0.25 × 0.15 × 0.10

Data collection		
Diffractometer	Bruker SMART APEX CCD	Bruker SMART APEX CCD
Absorption correction	Multi-scan (*SADABS*; Bruker, 2014 ▸)	Multi-scan (*SADABS*; Bruker, 2014 ▸)
*T*_min_, *T*_max_	0.636, 0.886	0.561, 0.780
No. of measured, independent and observed [*I* > 2σ(*I*)] reflections	13292, 8926, 8227	13359, 8915, 8062
*R*_int_	0.021	0.021
(sin θ/λ)_max_ (Å^−1^)	0.720	0.720

Refinement		
*R*[*F* ^2^ > 2σ(*F* ^2^)], *wR*(*F* ^2^), *S*	0.029, 0.069, 1.03	0.030, 0.073, 1.04
No. of reflections	8926	8915
No. of parameters	429	429
No. of restraints	0	1
H-atom treatment	H atoms treated by a mixture of independent and constrained refinement	H atoms treated by a mixture of independent and constrained refinement
Δρ_max_, Δρ_min_ (e Å^−3^)	1.48, −0.73	1.73, −0.79

Computer programs: *SMART* and *SAINT* (Bruker, 2014 ▸), *SHELXT2014* (Sheldrick, 2015*a* ▸), *SHELXL2014* (Sheldrick, 2015*b* ▸), *SHELXTL* (Sheldrick, 2008 ▸) and *Mercury* (Macrae *et al.*, 2008 ▸).

## supplementary crystallographic information

### Triaqua[2,6-diacetylpyridine bis(benzoylhydrazone)]methanolterbium(III) trichloride methanol disolvate (TbDAPBH2) Crystal data

**Table:**

[Tb(C_23_H_21_N_5_O_2_)(CH_4_O)(H_2_O)_3_)]Cl_3_·2CH_4_O	*Z* = 2
*M**_r_* = 814.89	*F*(000) = 820
Triclinic, *P*1	*D*_x_ = 1.673 Mg m^−^^3^
*a* = 8.9703 (7) Å	Mo *K*α radiation, λ = 0.71073 Å
*b* = 12.6433 (9) Å	Cell parameters from 7626 reflections
*c* = 14.4233 (11) Å	θ = 2.3–30.4°
α = 87.004 (1)°	µ = 2.49 mm^−^^1^
β = 88.752 (1)°	*T* = 100 K
γ = 81.980 (1)°	Plate, colorless
*V* = 1617.4 (2) Å^3^	0.20 × 0.15 × 0.05 mm

### Triaqua[2,6-diacetylpyridine bis(benzoylhydrazone)]methanolterbium(III) trichloride methanol disolvate (TbDAPBH2) Data collection

**Table:**

Bruker SMART APEX CCD diffractometer	8926 independent reflections
Radiation source: sealed tube	8227 reflections with *I* > 2σ(*I*)
Detector resolution: 8.366 pixels mm^-1^	*R*_int_ = 0.021
phi and ω scans	θ_max_ = 30.8°, θ_min_ = 2.1°
Absorption correction: multi-scan (SADABS; Bruker, 2014)	*h* = −12→12
*T*_min_ = 0.636, *T*_max_ = 0.886	*k* = −12→17
13292 measured reflections	*l* = −16→20

### Triaqua[2,6-diacetylpyridine bis(benzoylhydrazone)]methanolterbium(III) trichloride methanol disolvate (TbDAPBH2) Refinement

**Table:**

Refinement on *F*^2^	Primary atom site location: structure-invariant direct methods
Least-squares matrix: full	Secondary atom site location: difference Fourier map
*R*[*F*^2^ > 2σ(*F*^2^)] = 0.029	Hydrogen site location: mixed
*wR*(*F*^2^) = 0.069	H atoms treated by a mixture of independent and constrained refinement
*S* = 1.03	*w* = 1/[σ^2^(*F*_o_^2^) + (0.0355*P*)^2^ + 0.3922*P*] where *P* = (*F*_o_^2^ + 2*F*_c_^2^)/3
8926 reflections	(Δ/σ)_max_ = 0.001
429 parameters	Δρ_max_ = 1.48 e Å^−^^3^
0 restraints	Δρ_min_ = −0.73 e Å^−^^3^

### Triaqua[2,6-diacetylpyridine bis(benzoylhydrazone)]methanolterbium(III) trichloride methanol disolvate (TbDAPBH2) Special details

**Table:**

Geometry. All esds (except the esd in the dihedral angle between two l.s. planes) are estimated using the full covariance matrix. The cell esds are taken into account individually in the estimation of esds in distances, angles and torsion angles; correlations between esds in cell parameters are only used when they are defined by crystal symmetry. An approximate (isotropic) treatment of cell esds is used for estimating esds involving l.s. planes.

### Triaqua[2,6-diacetylpyridine bis(benzoylhydrazone)]methanolterbium(III) trichloride methanol disolvate (TbDAPBH2) Fractional atomic coordinates and isotropic or equivalent isotropic displacement parameters (Å^2^)

**Table:**

	*x*	*y*	*z*	*U*_iso_*/*U*_eq_	
C1	0.3931 (3)	−0.18034 (19)	0.27986 (17)	0.0141 (4)	
C2	0.4840 (3)	−0.27866 (19)	0.31946 (17)	0.0145 (5)	
C3	0.6288 (3)	−0.2707 (2)	0.34747 (19)	0.0185 (5)	
H3	0.6690	−0.2055	0.3365	0.022*	
C4	0.7149 (3)	−0.3579 (2)	0.3915 (2)	0.0238 (6)	
H4	0.8137	−0.3523	0.4113	0.029*	
C5	0.6563 (3)	−0.4533 (2)	0.4066 (2)	0.0232 (6)	
H5	0.7154	−0.5130	0.4367	0.028*	
C6	0.5125 (3)	−0.4623 (2)	0.3781 (2)	0.0212 (5)	
H6	0.4733	−0.5280	0.3883	0.025*	
C7	0.4255 (3)	−0.3752 (2)	0.33454 (19)	0.0186 (5)	
H7	0.3266	−0.3810	0.3151	0.022*	
C8	0.0392 (3)	−0.08870 (19)	0.18845 (17)	0.0133 (4)	
C9	−0.0525 (3)	−0.1790 (2)	0.2001 (2)	0.0187 (5)	
H9A	−0.0136	−0.2273	0.2521	0.028*	
H9B	−0.0461	−0.2183	0.1431	0.028*	
H9C	−0.1578	−0.1504	0.2129	0.028*	
C10	−0.0267 (3)	0.01568 (19)	0.14391 (17)	0.0136 (4)	
C11	−0.1615 (3)	0.0250 (2)	0.09601 (19)	0.0183 (5)	
H11	−0.2197	−0.0323	0.0976	0.022*	
C12	−0.2087 (3)	0.1203 (2)	0.0458 (2)	0.0202 (5)	
H12	−0.3010	0.1296	0.0134	0.024*	
C13	−0.1197 (3)	0.2013 (2)	0.04380 (18)	0.0171 (5)	
H13	−0.1484	0.2661	0.0084	0.021*	
C14	0.0129 (3)	0.18641 (18)	0.09447 (17)	0.0127 (4)	
C15	0.1148 (3)	0.26892 (18)	0.09226 (17)	0.0127 (4)	
C16	0.0854 (3)	0.36814 (19)	0.03070 (19)	0.0187 (5)	
H16A	0.1554	0.3628	−0.0225	0.028*	
H16B	0.0998	0.4302	0.0657	0.028*	
H16C	−0.0183	0.3765	0.0086	0.028*	
C17	0.4647 (3)	0.28258 (18)	0.18861 (17)	0.0136 (4)	
C18	0.5814 (3)	0.35476 (19)	0.18763 (18)	0.0162 (5)	
C19	0.5606 (3)	0.4577 (2)	0.1460 (2)	0.0205 (5)	
H19	0.4690	0.4836	0.1154	0.025*	
C20	0.6730 (3)	0.5226 (2)	0.1491 (2)	0.0233 (6)	
H20	0.6579	0.5932	0.1218	0.028*	
C21	0.8076 (3)	0.4835 (2)	0.1924 (2)	0.0237 (6)	
H21	0.8854	0.5273	0.1938	0.028*	
C22	0.8297 (3)	0.3809 (2)	0.2338 (2)	0.0225 (5)	
H22	0.9222	0.3548	0.2632	0.027*	
C23	0.7164 (3)	0.3164 (2)	0.23211 (19)	0.0185 (5)	
H23	0.7308	0.2466	0.2611	0.022*	
C24	0.4784 (3)	0.1550 (2)	0.4460 (2)	0.0232 (6)	
H24A	0.3820	0.1904	0.4698	0.035*	
H24B	0.5282	0.2067	0.4080	0.035*	
H24C	0.5427	0.1268	0.4981	0.035*	
C25	0.9063 (3)	0.5147 (2)	0.6159 (2)	0.0274 (6)	
H25A	0.9243	0.5643	0.5637	0.041*	
H25B	0.8624	0.4544	0.5930	0.041*	
H25C	1.0018	0.4881	0.6463	0.041*	
C26	0.1290 (3)	0.7633 (2)	0.5060 (2)	0.0292 (6)	
H26A	0.1504	0.8287	0.5337	0.044*	
H26B	0.1736	0.7594	0.4435	0.044*	
H26C	0.1722	0.7009	0.5446	0.044*	
Cl1	0.58645 (7)	0.81886 (5)	0.06470 (4)	0.01793 (12)	
Cl2	0.18932 (7)	0.59026 (5)	0.15256 (5)	0.02071 (13)	
Cl3	0.18676 (7)	0.02722 (5)	0.60038 (5)	0.02070 (13)	
H3S	0.517 (5)	0.035 (4)	0.383 (3)	0.059 (15)*	
H7S	0.804 (4)	0.535 (3)	0.722 (2)	0.020 (9)*	
H8S	−0.061 (5)	0.822 (3)	0.462 (3)	0.055 (13)*	
H4SA	0.179 (5)	0.011 (3)	0.421 (3)	0.044 (14)*	
H5SA	0.127 (4)	0.217 (3)	0.377 (3)	0.036 (11)*	
H6SA	0.445 (4)	−0.021 (3)	0.103 (3)	0.035 (11)*	
H4SB	0.063 (4)	0.003 (3)	0.378 (3)	0.034 (10)*	
H5SB	0.188 (4)	0.283 (3)	0.323 (3)	0.045 (12)*	
H6SB	0.404 (4)	0.070 (3)	0.068 (3)	0.021 (9)*	
N1	0.2562 (2)	−0.18805 (16)	0.24489 (15)	0.0153 (4)	
H1A	0.2202	−0.2492	0.2433	0.018*	
N2	0.1773 (2)	−0.09152 (15)	0.21167 (14)	0.0126 (4)	
N3	0.0574 (2)	0.09586 (15)	0.14648 (14)	0.0124 (4)	
N4	0.2332 (2)	0.24464 (15)	0.14258 (14)	0.0117 (4)	
N5	0.3365 (2)	0.31596 (16)	0.14172 (15)	0.0152 (4)	
H5A	0.3200	0.3792	0.1125	0.018*	
O1	0.43783 (19)	−0.09181 (13)	0.28311 (13)	0.0153 (3)	
O2	0.48215 (19)	0.19393 (14)	0.23100 (13)	0.0174 (4)	
O3	0.4522 (2)	0.06898 (15)	0.39042 (13)	0.0160 (4)	
O4	0.1509 (2)	0.00969 (16)	0.38079 (15)	0.0180 (4)	
O5	0.1709 (2)	0.22339 (15)	0.33114 (14)	0.0178 (4)	
O6	0.4055 (2)	0.03235 (16)	0.10887 (15)	0.0207 (4)	
O7	0.8055 (2)	0.56858 (16)	0.68054 (16)	0.0266 (5)	
O8	−0.0300 (2)	0.76493 (16)	0.50082 (15)	0.0250 (4)	
Tb1	0.29292 (2)	0.07670 (2)	0.25062 (2)	0.01026 (4)	

### Triaqua[2,6-diacetylpyridine bis(benzoylhydrazone)]methanolterbium(III) trichloride methanol disolvate (TbDAPBH2) Atomic displacement parameters (Å^2^)

**Table:**

	*U*^11^	*U*^22^	*U*^33^	*U*^12^	*U*^13^	*U*^23^
C1	0.0155 (11)	0.0136 (11)	0.0133 (11)	−0.0022 (8)	0.0007 (9)	−0.0008 (8)
C2	0.0168 (11)	0.0127 (10)	0.0137 (12)	−0.0008 (8)	−0.0007 (9)	−0.0004 (8)
C3	0.0171 (12)	0.0157 (11)	0.0225 (14)	−0.0015 (9)	−0.0025 (10)	0.0005 (9)
C4	0.0174 (12)	0.0234 (13)	0.0302 (16)	0.0006 (10)	−0.0073 (11)	−0.0029 (11)
C5	0.0251 (14)	0.0204 (13)	0.0223 (14)	0.0033 (10)	−0.0060 (11)	0.0020 (10)
C6	0.0262 (14)	0.0153 (12)	0.0218 (14)	−0.0034 (10)	−0.0033 (11)	0.0032 (10)
C7	0.0199 (12)	0.0163 (11)	0.0197 (13)	−0.0038 (9)	−0.0028 (10)	0.0025 (9)
C8	0.0143 (11)	0.0136 (10)	0.0124 (11)	−0.0031 (8)	−0.0017 (9)	−0.0010 (8)
C9	0.0161 (11)	0.0159 (11)	0.0251 (14)	−0.0061 (9)	−0.0023 (10)	0.0006 (10)
C10	0.0119 (10)	0.0148 (11)	0.0138 (12)	−0.0010 (8)	−0.0010 (9)	−0.0001 (8)
C11	0.0160 (12)	0.0158 (11)	0.0242 (14)	−0.0056 (9)	−0.0049 (10)	0.0001 (10)
C12	0.0145 (11)	0.0215 (12)	0.0254 (14)	−0.0049 (9)	−0.0075 (10)	0.0011 (10)
C13	0.0154 (11)	0.0167 (11)	0.0189 (13)	−0.0016 (9)	−0.0047 (9)	0.0025 (9)
C14	0.0125 (10)	0.0135 (10)	0.0124 (11)	−0.0022 (8)	−0.0014 (9)	−0.0003 (8)
C15	0.0152 (11)	0.0115 (10)	0.0112 (11)	−0.0008 (8)	−0.0004 (9)	−0.0005 (8)
C16	0.0209 (12)	0.0148 (11)	0.0205 (13)	−0.0038 (9)	−0.0066 (10)	0.0050 (9)
C17	0.0111 (10)	0.0140 (11)	0.0155 (12)	−0.0014 (8)	0.0023 (9)	0.0006 (8)
C18	0.0130 (11)	0.0165 (11)	0.0192 (13)	−0.0032 (9)	0.0009 (9)	−0.0004 (9)
C19	0.0154 (12)	0.0198 (12)	0.0262 (15)	−0.0042 (9)	0.0001 (10)	0.0044 (10)
C20	0.0219 (13)	0.0185 (12)	0.0303 (16)	−0.0082 (10)	0.0037 (11)	0.0042 (11)
C21	0.0206 (13)	0.0243 (13)	0.0289 (15)	−0.0119 (10)	0.0050 (11)	−0.0040 (11)
C22	0.0157 (12)	0.0260 (13)	0.0267 (15)	−0.0047 (10)	−0.0031 (10)	−0.0035 (11)
C23	0.0181 (12)	0.0190 (12)	0.0189 (13)	−0.0039 (9)	−0.0005 (10)	−0.0004 (9)
C24	0.0266 (14)	0.0210 (13)	0.0230 (14)	−0.0045 (10)	−0.0070 (11)	−0.0047 (10)
C25	0.0277 (15)	0.0273 (14)	0.0280 (16)	−0.0054 (11)	0.0015 (12)	−0.0058 (12)
C26	0.0270 (15)	0.0222 (14)	0.0377 (18)	−0.0024 (11)	0.0050 (13)	−0.0004 (12)
Cl1	0.0197 (3)	0.0156 (3)	0.0175 (3)	−0.0001 (2)	0.0012 (2)	0.0021 (2)
Cl2	0.0225 (3)	0.0190 (3)	0.0211 (3)	−0.0038 (2)	−0.0071 (2)	0.0001 (2)
Cl3	0.0164 (3)	0.0262 (3)	0.0203 (3)	−0.0051 (2)	−0.0007 (2)	−0.0022 (2)
N1	0.0158 (10)	0.0113 (9)	0.0185 (11)	−0.0019 (7)	−0.0043 (8)	0.0029 (8)
N2	0.0155 (9)	0.0094 (9)	0.0130 (10)	−0.0019 (7)	−0.0054 (8)	0.0010 (7)
N3	0.0130 (9)	0.0118 (9)	0.0119 (10)	−0.0014 (7)	−0.0011 (7)	0.0015 (7)
N4	0.0109 (9)	0.0123 (9)	0.0127 (10)	−0.0044 (7)	−0.0011 (7)	0.0001 (7)
N5	0.0143 (10)	0.0131 (9)	0.0186 (11)	−0.0053 (7)	−0.0033 (8)	0.0044 (8)
O1	0.0165 (8)	0.0101 (8)	0.0199 (9)	−0.0033 (6)	−0.0035 (7)	−0.0004 (6)
O2	0.0148 (8)	0.0149 (8)	0.0222 (10)	−0.0037 (6)	−0.0032 (7)	0.0061 (7)
O3	0.0161 (9)	0.0166 (9)	0.0154 (9)	−0.0018 (7)	−0.0042 (7)	−0.0019 (7)
O4	0.0172 (10)	0.0227 (10)	0.0155 (10)	−0.0082 (7)	−0.0003 (8)	−0.0003 (7)
O5	0.0225 (9)	0.0137 (9)	0.0164 (10)	−0.0010 (7)	0.0055 (8)	0.0001 (7)
O6	0.0290 (11)	0.0130 (9)	0.0180 (10)	0.0023 (8)	0.0052 (8)	0.0030 (8)
O7	0.0355 (12)	0.0171 (9)	0.0272 (12)	−0.0061 (8)	0.0056 (9)	0.0039 (8)
O8	0.0275 (10)	0.0252 (10)	0.0230 (11)	−0.0079 (8)	0.0033 (8)	0.0012 (8)
Tb1	0.01062 (6)	0.00929 (6)	0.01094 (6)	−0.00193 (4)	−0.00114 (4)	0.00083 (4)

### Triaqua[2,6-diacetylpyridine bis(benzoylhydrazone)]methanolterbium(III) trichloride methanol disolvate (TbDAPBH2) Geometric parameters (Å, º)

**Table:**

C1—O1	1.244 (3)	C20—C21	1.388 (4)
C1—N1	1.355 (3)	C20—H20	0.9500
C1—C2	1.483 (3)	C21—C22	1.390 (4)
C2—C3	1.387 (4)	C21—H21	0.9500
C2—C7	1.399 (3)	C22—C23	1.390 (4)
C3—C4	1.388 (4)	C22—H22	0.9500
C3—H3	0.9500	C23—H23	0.9500
C4—C5	1.387 (4)	C24—O3	1.432 (3)
C4—H4	0.9500	C24—H24A	0.9800
C5—C6	1.384 (4)	C24—H24B	0.9800
C5—H5	0.9500	C24—H24C	0.9800
C6—C7	1.388 (3)	C25—O7	1.417 (4)
C6—H6	0.9500	C25—H25A	0.9800
C7—H7	0.9500	C25—H25B	0.9800
C8—N2	1.286 (3)	C25—H25C	0.9800
C8—C10	1.488 (3)	C26—O8	1.427 (4)
C8—C9	1.498 (3)	C26—H26A	0.9800
C9—H9A	0.9800	C26—H26B	0.9800
C9—H9B	0.9800	C26—H26C	0.9800
C9—H9C	0.9800	N1—N2	1.390 (3)
C10—N3	1.348 (3)	N1—H1A	0.8800
C10—C11	1.393 (3)	N2—Tb1	2.5845 (19)
C11—C12	1.390 (4)	N3—Tb1	2.596 (2)
C11—H11	0.9500	N4—N5	1.380 (3)
C12—C13	1.383 (4)	N4—Tb1	2.5685 (19)
C12—H12	0.9500	N5—H5A	0.8800
C13—C14	1.394 (3)	O1—Tb1	2.3660 (16)
C13—H13	0.9500	O2—Tb1	2.4074 (17)
C14—N3	1.352 (3)	O3—Tb1	2.4867 (18)
C14—C15	1.479 (3)	O3—H3S	0.68 (4)
C15—N4	1.291 (3)	O4—Tb1	2.428 (2)
C15—C16	1.494 (3)	O4—H4SA	0.64 (5)
C16—H16A	0.9800	O4—H4SB	0.80 (4)
C16—H16B	0.9800	O5—Tb1	2.3642 (19)
C16—H16C	0.9800	O5—H5SA	0.78 (4)
C17—O2	1.240 (3)	O5—H5SB	0.80 (4)
C17—N5	1.351 (3)	O6—Tb1	2.321 (2)
C17—C18	1.481 (3)	O6—H6SA	0.73 (4)
C18—C19	1.395 (3)	O6—H6SB	0.74 (4)
C18—C23	1.397 (4)	O7—H7S	0.72 (3)
C19—C20	1.389 (4)	O8—H8S	0.91 (4)
C19—H19	0.9500		
			
O1—C1—N1	120.6 (2)	H24B—C24—H24C	109.5
O1—C1—C2	120.8 (2)	O7—C25—H25A	109.5
N1—C1—C2	118.6 (2)	O7—C25—H25B	109.5
C3—C2—C7	119.9 (2)	H25A—C25—H25B	109.5
C3—C2—C1	117.5 (2)	O7—C25—H25C	109.5
C7—C2—C1	122.4 (2)	H25A—C25—H25C	109.5
C2—C3—C4	120.1 (2)	H25B—C25—H25C	109.5
C2—C3—H3	120.0	O8—C26—H26A	109.5
C4—C3—H3	120.0	O8—C26—H26B	109.5
C5—C4—C3	119.8 (3)	H26A—C26—H26B	109.5
C5—C4—H4	120.1	O8—C26—H26C	109.5
C3—C4—H4	120.1	H26A—C26—H26C	109.5
C6—C5—C4	120.5 (2)	H26B—C26—H26C	109.5
C6—C5—H5	119.7	C1—N1—N2	114.57 (19)
C4—C5—H5	119.7	C1—N1—H1A	122.7
C5—C6—C7	119.9 (2)	N2—N1—H1A	122.7
C5—C6—H6	120.0	C8—N2—N1	118.8 (2)
C7—C6—H6	120.0	C8—N2—Tb1	123.18 (15)
C6—C7—C2	119.7 (2)	N1—N2—Tb1	115.06 (14)
C6—C7—H7	120.1	C10—N3—C14	117.6 (2)
C2—C7—H7	120.1	C10—N3—Tb1	120.64 (15)
N2—C8—C10	113.4 (2)	C14—N3—Tb1	121.71 (15)
N2—C8—C9	126.2 (2)	C15—N4—N5	118.17 (19)
C10—C8—C9	120.4 (2)	C15—N4—Tb1	126.08 (15)
C8—C9—H9A	109.5	N5—N4—Tb1	115.71 (14)
C8—C9—H9B	109.5	C17—N5—N4	115.84 (19)
H9A—C9—H9B	109.5	C17—N5—H5A	122.1
C8—C9—H9C	109.5	N4—N5—H5A	122.1
H9A—C9—H9C	109.5	C1—O1—Tb1	126.00 (15)
H9B—C9—H9C	109.5	C17—O2—Tb1	125.09 (15)
N3—C10—C11	123.1 (2)	C24—O3—Tb1	128.24 (16)
N3—C10—C8	116.1 (2)	C24—O3—H3S	112 (4)
C11—C10—C8	120.6 (2)	Tb1—O3—H3S	108 (4)
C12—C11—C10	118.4 (2)	Tb1—O4—H4SA	116 (4)
C12—C11—H11	120.8	Tb1—O4—H4SB	123 (3)
C10—C11—H11	120.8	H4SA—O4—H4SB	118 (5)
C13—C12—C11	119.2 (2)	Tb1—O5—H5SA	123 (3)
C13—C12—H12	120.4	Tb1—O5—H5SB	125 (3)
C11—C12—H12	120.4	H5SA—O5—H5SB	110 (4)
C12—C13—C14	119.0 (2)	Tb1—O6—H6SA	120 (3)
C12—C13—H13	120.5	Tb1—O6—H6SB	124 (3)
C14—C13—H13	120.5	H6SA—O6—H6SB	116 (4)
N3—C14—C13	122.5 (2)	C25—O7—H7S	109 (3)
N3—C14—C15	116.2 (2)	C26—O8—H8S	105 (3)
C13—C14—C15	121.3 (2)	O6—Tb1—O5	142.11 (7)
N4—C15—C14	114.7 (2)	O6—Tb1—O1	76.15 (7)
N4—C15—C16	123.8 (2)	O5—Tb1—O1	139.14 (7)
C14—C15—C16	121.4 (2)	O6—Tb1—O2	76.48 (7)
C15—C16—H16A	109.5	O5—Tb1—O2	81.03 (7)
C15—C16—H16B	109.5	O1—Tb1—O2	102.60 (6)
H16A—C16—H16B	109.5	O6—Tb1—O4	143.61 (7)
C15—C16—H16C	109.5	O5—Tb1—O4	71.13 (7)
H16A—C16—H16C	109.5	O1—Tb1—O4	79.33 (7)
H16B—C16—H16C	109.5	O2—Tb1—O4	135.63 (7)
O2—C17—N5	120.2 (2)	O6—Tb1—O3	119.22 (7)
O2—C17—C18	121.8 (2)	O5—Tb1—O3	78.63 (7)
N5—C17—C18	118.1 (2)	O1—Tb1—O3	65.79 (6)
C19—C18—C23	119.8 (2)	O2—Tb1—O3	68.37 (6)
C19—C18—C17	123.0 (2)	O4—Tb1—O3	72.66 (7)
C23—C18—C17	117.2 (2)	O6—Tb1—N4	74.40 (7)
C20—C19—C18	120.3 (2)	O5—Tb1—N4	68.22 (7)
C20—C19—H19	119.8	O1—Tb1—N4	149.40 (6)
C18—C19—H19	119.8	O2—Tb1—N4	62.41 (6)
C21—C20—C19	119.5 (2)	O4—Tb1—N4	130.58 (7)
C21—C20—H20	120.2	O3—Tb1—N4	123.50 (6)
C19—C20—H20	120.2	O6—Tb1—N2	76.97 (7)
C20—C21—C22	120.6 (2)	O5—Tb1—N2	126.12 (7)
C20—C21—H21	119.7	O1—Tb1—N2	62.47 (6)
C22—C21—H21	119.7	O2—Tb1—N2	152.08 (7)
C23—C22—C21	120.0 (3)	O4—Tb1—N2	67.93 (7)
C23—C22—H22	120.0	O3—Tb1—N2	118.85 (6)
C21—C22—H22	120.0	N4—Tb1—N2	117.64 (6)
C22—C23—C18	119.7 (2)	O6—Tb1—N3	79.68 (7)
C22—C23—H23	120.1	O5—Tb1—N3	87.34 (7)
C18—C23—H23	120.1	O1—Tb1—N3	121.25 (6)
O3—C24—H24A	109.5	O2—Tb1—N3	122.36 (6)
O3—C24—H24B	109.5	O4—Tb1—N3	90.74 (7)
H24A—C24—H24B	109.5	O3—Tb1—N3	160.96 (6)
O3—C24—H24C	109.5	N4—Tb1—N3	60.86 (6)
H24A—C24—H24C	109.5	N2—Tb1—N3	60.23 (6)

### Triaqua[2,6-diacetylpyridine bis(benzoylhydrazone)]methanolterbium(III) trichloride methanol disolvate (TbDAPBH2) Hydrogen-bond geometry (Å, º)

**Table:**

*D*—H···*A*	*D*—H	H···*A*	*D*···*A*	*D*—H···*A*
O4—H4*SA*···Cl3	0.64 (4)	2.61 (4)	3.213 (2)	159 (5)
N1—H1*A*···Cl2^i^	0.88	2.52	3.299 (2)	148
O6—H6*SA*···Cl1^i^	0.73 (4)	2.32 (4)	3.040 (2)	172 (4)
O3—H3*S*···Cl3^ii^	0.68 (5)	2.68 (5)	3.2998 (19)	153 (5)
O4—H4*SB*···Cl3^iii^	0.81 (4)	2.34 (4)	3.1323 (19)	169 (4)
O6—H6*SB*···Cl1^iv^	0.74 (4)	2.32 (4)	3.058 (2)	176 (3)
O7—H7*S*···Cl2^v^	0.72 (3)	2.34 (3)	3.050 (2)	174 (4)
O8—H8*S*···Cl3^vi^	0.91 (4)	2.23 (4)	3.110 (2)	163 (4)
O5—H5*SA*···O8^vi^	0.77 (4)	1.96 (4)	2.710 (3)	166 (4)
O5—H5*SB*···O7^v^	0.79 (4)	1.88 (4)	2.664 (3)	168 (4)
C7—H7···Cl2^i^	0.95	2.74	3.491 (3)	137
C11—H11···Cl1^vii^	0.95	2.80	3.731 (3)	167
C12—H12···Cl1^viii^	0.95	2.80	3.741 (3)	172
C16—H16*B*···Cl2	0.98	2.66	3.628 (3)	170
C16—H16*C*···Cl2^viii^	0.98	2.79	3.621 (3)	143
C19—H19···Cl2	0.95	2.73	3.515 (3)	140
C26—H26*A*···Cl3^ix^	0.98	2.80	3.774 (3)	174
C4—H4···O8^x^	0.95	2.59	3.397 (3)	143

Symmetry codes: (i) *x*, *y*−1, *z*; (ii) −*x*+1, −*y*, −*z*+1; (iii) −*x*, −*y*, −*z*+1; (iv) −*x*+1, −*y*+1, −*z*; (v) −*x*+1, −*y*+1, −*z*+1; (vi) −*x*, −*y*+1, −*z*+1; (vii) *x*−1, *y*−1, *z*; (viii) −*x*, −*y*+1, −*z*; (ix) *x*, *y*+1, *z*; (x) *x*+1, *y*−1, *z*.

### Triaqua[2,6-diacetylpyridine bis(benzoylhydrazone)]methanoldysprosium(III) trichloride methanol disolvate (DyDAPBH2) Crystal data

**Table:**

[Dy(C_23_H_21_N_5_O_2_)(CH_4_O)(H_2_O)_3_)]Cl_3_·2CH_4_O	*Z* = 2
*M**_r_* = 818.47	*F*(000) = 822
Triclinic, *P*1	*D*_x_ = 1.684 Mg m^−^^3^
*a* = 8.9852 (7) Å	Mo *K*α radiation, λ = 0.71073 Å
*b* = 12.6242 (10) Å	Cell parameters from 7204 reflections
*c* = 14.3887 (12) Å	θ = 2.2–30.5°
α = 87.062 (1)°	µ = 2.62 mm^−^^1^
β = 88.810 (1)°	*T* = 100 K
γ = 82.068 (1)°	Plate, colorless
*V* = 1614.2 (2) Å^3^	0.25 × 0.15 × 0.10 mm

### Triaqua[2,6-diacetylpyridine bis(benzoylhydrazone)]methanoldysprosium(III) trichloride methanol disolvate (DyDAPBH2) Data collection

**Table:**

Bruker SMART APEX CCD diffractometer	8915 independent reflections
Radiation source: sealed tube	8062 reflections with *I* > 2σ(*I*)
Detector resolution: 8.366 pixels mm^-1^	*R*_int_ = 0.021
phi and ω scans	θ_max_ = 30.8°, θ_min_ = 2.2°
Absorption correction: multi-scan (SADABS; Bruker, 2014)	*h* = −12→9
*T*_min_ = 0.561, *T*_max_ = 0.780	*k* = −17→18
13359 measured reflections	*l* = −20→17

### Triaqua[2,6-diacetylpyridine bis(benzoylhydrazone)]methanoldysprosium(III) trichloride methanol disolvate (DyDAPBH2) Refinement

**Table:**

Refinement on *F*^2^	Primary atom site location: structure-invariant direct methods
Least-squares matrix: full	Secondary atom site location: difference Fourier map
*R*[*F*^2^ > 2σ(*F*^2^)] = 0.030	Hydrogen site location: mixed
*wR*(*F*^2^) = 0.073	H atoms treated by a mixture of independent and constrained refinement
*S* = 1.04	*w* = 1/[σ^2^(*F*_o_^2^) + (0.0355*P*)^2^ + 0.3922*P*] where *P* = (*F*_o_^2^ + 2*F*_c_^2^)/3
8915 reflections	(Δ/σ)_max_ = 0.001
429 parameters	Δρ_max_ = 1.73 e Å^−^^3^
1 restraint	Δρ_min_ = −0.79 e Å^−^^3^

### Triaqua[2,6-diacetylpyridine bis(benzoylhydrazone)]methanoldysprosium(III) trichloride methanol disolvate (DyDAPBH2) Special details

**Table:**

Geometry. All esds (except the esd in the dihedral angle between two l.s. planes) are estimated using the full covariance matrix. The cell esds are taken into account individually in the estimation of esds in distances, angles and torsion angles; correlations between esds in cell parameters are only used when they are defined by crystal symmetry. An approximate (isotropic) treatment of cell esds is used for estimating esds involving l.s. planes.

### Triaqua[2,6-diacetylpyridine bis(benzoylhydrazone)]methanoldysprosium(III) trichloride methanol disolvate (DyDAPBH2) Fractional atomic coordinates and isotropic or equivalent isotropic displacement parameters (Å^2^)

**Table:**

	*x*	*y*	*z*	*U*_iso_*/*U*_eq_	
C1	0.3923 (3)	−0.1799 (2)	0.27938 (19)	0.0130 (5)	
C2	0.4831 (3)	−0.2781 (2)	0.3189 (2)	0.0138 (5)	
C3	0.6283 (3)	−0.2700 (2)	0.3466 (2)	0.0184 (6)	
H3	0.6686	−0.2047	0.3354	0.022*	
C4	0.7142 (4)	−0.3572 (2)	0.3906 (2)	0.0242 (7)	
H4	0.8129	−0.3516	0.4102	0.029*	
C5	0.6554 (4)	−0.4525 (2)	0.4057 (2)	0.0237 (7)	
H5	0.7141	−0.5122	0.4361	0.028*	
C6	0.5134 (4)	−0.4617 (2)	0.3774 (2)	0.0213 (6)	
H6	0.4748	−0.5278	0.3869	0.026*	
C7	0.4259 (3)	−0.3743 (2)	0.3347 (2)	0.0183 (6)	
H7	0.3266	−0.3802	0.3163	0.022*	
C8	0.0387 (3)	−0.0891 (2)	0.1879 (2)	0.0128 (5)	
C9	−0.0519 (3)	−0.1800 (2)	0.1998 (2)	0.0192 (6)	
H9A	−0.0096	−0.2300	0.2499	0.029*	
H9B	−0.0494	−0.2174	0.1417	0.029*	
H9C	−0.1561	−0.1521	0.2157	0.029*	
C10	−0.0273 (3)	0.0146 (2)	0.1440 (2)	0.0137 (5)	
C11	−0.1621 (3)	0.0250 (2)	0.0962 (2)	0.0191 (6)	
H11	−0.2207	−0.0322	0.0979	0.023*	
C12	−0.2094 (3)	0.1199 (2)	0.0463 (2)	0.0202 (6)	
H12	−0.3020	0.1293	0.0142	0.024*	
C13	−0.1196 (3)	0.2009 (2)	0.0437 (2)	0.0178 (6)	
H13	−0.1474	0.2654	0.0075	0.021*	
C14	0.0122 (3)	0.1862 (2)	0.0949 (2)	0.0131 (5)	
C15	0.1153 (3)	0.2686 (2)	0.09186 (19)	0.0125 (5)	
C16	0.0853 (3)	0.3676 (2)	0.0306 (2)	0.0185 (6)	
H16A	0.1581	0.3641	−0.0212	0.028*	
H16B	0.0947	0.4301	0.0666	0.028*	
H16C	−0.0167	0.3737	0.0061	0.028*	
C17	0.4641 (3)	0.2816 (2)	0.1881 (2)	0.0137 (5)	
C18	0.5818 (3)	0.3531 (2)	0.1874 (2)	0.0156 (5)	
C19	0.5607 (3)	0.4565 (2)	0.1457 (2)	0.0208 (6)	
H19	0.4693	0.4824	0.1150	0.025*	
C20	0.6734 (4)	0.5213 (3)	0.1491 (2)	0.0241 (7)	
H20	0.6586	0.5921	0.1220	0.029*	
C21	0.8072 (4)	0.4819 (3)	0.1924 (2)	0.0231 (7)	
H21	0.8852	0.5255	0.1938	0.028*	
C22	0.8286 (3)	0.3798 (3)	0.2334 (2)	0.0225 (6)	
H22	0.9212	0.3538	0.2627	0.027*	
C23	0.7156 (3)	0.3146 (2)	0.2322 (2)	0.0179 (6)	
H23	0.7297	0.2450	0.2616	0.022*	
C24	0.4765 (4)	0.1551 (3)	0.4457 (2)	0.0251 (7)	
H24A	0.3806	0.1913	0.4692	0.038*	
H24B	0.5274	0.2064	0.4078	0.038*	
H24C	0.5401	0.1266	0.4982	0.038*	
C25	0.9067 (4)	0.5157 (3)	0.6162 (3)	0.0275 (7)	
H25A	0.9237	0.5649	0.5634	0.041*	
H25B	0.8635	0.4546	0.5940	0.041*	
H25C	1.0026	0.4901	0.6465	0.041*	
C26	0.1282 (4)	0.7637 (3)	0.5053 (3)	0.0318 (8)	
H26A	0.1511	0.8275	0.5350	0.048*	
H26B	0.1705	0.7626	0.4419	0.048*	
H26C	0.1721	0.6994	0.5413	0.048*	
Cl1	0.58557 (8)	0.81884 (5)	0.06449 (5)	0.01935 (14)	
Cl2	0.18964 (8)	0.58963 (6)	0.15245 (5)	0.02133 (15)	
Cl3	0.18672 (8)	0.02676 (6)	0.60011 (6)	0.02261 (15)	
Dy1	0.29156 (2)	0.07637 (2)	0.24991 (2)	0.01098 (4)	
H3S	0.525 (3)	0.029 (3)	0.387 (3)	0.038 (12)*	
H7S	0.804 (4)	0.539 (3)	0.729 (3)	0.024 (11)*	
H8S	−0.063 (6)	0.819 (4)	0.476 (4)	0.058 (17)*	
H4SA	0.167 (5)	0.019 (3)	0.431 (3)	0.028 (11)*	
H5SA	0.126 (5)	0.206 (3)	0.377 (3)	0.034 (12)*	
H6SA	0.452 (4)	−0.024 (3)	0.106 (3)	0.025 (11)*	
H4SB	0.069 (5)	0.011 (3)	0.379 (3)	0.026 (11)*	
H5SB	0.190 (6)	0.276 (4)	0.327 (4)	0.061 (17)*	
H6SB	0.406 (4)	0.064 (3)	0.071 (2)	0.006 (9)*	
N1	0.2557 (3)	−0.18801 (18)	0.24483 (17)	0.0147 (5)	
H1A	0.2196	−0.2493	0.2440	0.018*	
N2	0.1772 (3)	−0.09208 (18)	0.21085 (17)	0.0142 (5)	
N3	0.0566 (3)	0.09572 (18)	0.14657 (17)	0.0126 (4)	
N4	0.2328 (3)	0.24374 (18)	0.14232 (17)	0.0132 (4)	
N5	0.3360 (3)	0.31481 (19)	0.14179 (18)	0.0152 (5)	
H5A	0.3192	0.3784	0.1129	0.018*	
O1	0.4364 (2)	−0.09136 (15)	0.28269 (15)	0.0154 (4)	
O2	0.4808 (2)	0.19244 (16)	0.23072 (15)	0.0182 (4)	
O3	0.4493 (2)	0.06843 (16)	0.38954 (15)	0.0168 (4)	
O4	0.1507 (3)	0.00937 (18)	0.38004 (17)	0.0188 (4)	
O5	0.1698 (3)	0.22245 (18)	0.33018 (16)	0.0188 (4)	
O6	0.4048 (3)	0.0315 (2)	0.10881 (18)	0.0217 (5)	
O7	0.8064 (3)	0.56962 (18)	0.6807 (2)	0.0279 (5)	
O8	−0.0309 (3)	0.7659 (2)	0.50160 (19)	0.0277 (5)	

### Triaqua[2,6-diacetylpyridine bis(benzoylhydrazone)]methanoldysprosium(III) trichloride methanol disolvate (DyDAPBH2) Atomic displacement parameters (Å^2^)

**Table:**

	*U*^11^	*U*^22^	*U*^33^	*U*^12^	*U*^13^	*U*^23^
C1	0.0137 (13)	0.0129 (12)	0.0126 (13)	−0.0026 (10)	0.0019 (10)	−0.0017 (10)
C2	0.0163 (13)	0.0100 (12)	0.0150 (13)	−0.0015 (10)	−0.0015 (10)	0.0002 (10)
C3	0.0185 (14)	0.0142 (13)	0.0232 (16)	−0.0042 (11)	−0.0026 (11)	−0.0003 (11)
C4	0.0193 (15)	0.0196 (15)	0.0332 (19)	0.0011 (12)	−0.0074 (13)	−0.0029 (13)
C5	0.0287 (17)	0.0162 (14)	0.0240 (17)	0.0048 (12)	−0.0069 (13)	0.0028 (12)
C6	0.0265 (16)	0.0132 (13)	0.0242 (17)	−0.0038 (11)	0.0000 (12)	0.0030 (12)
C7	0.0190 (14)	0.0170 (14)	0.0201 (15)	−0.0067 (11)	−0.0032 (11)	0.0007 (11)
C8	0.0128 (13)	0.0104 (12)	0.0152 (13)	−0.0015 (9)	0.0006 (10)	−0.0021 (10)
C9	0.0160 (14)	0.0136 (13)	0.0289 (17)	−0.0064 (11)	0.0007 (12)	−0.0001 (12)
C10	0.0126 (13)	0.0128 (12)	0.0161 (14)	−0.0031 (10)	−0.0013 (10)	−0.0014 (10)
C11	0.0167 (14)	0.0154 (13)	0.0261 (16)	−0.0050 (11)	−0.0046 (11)	−0.0012 (12)
C12	0.0146 (14)	0.0200 (14)	0.0265 (17)	−0.0038 (11)	−0.0063 (11)	0.0009 (12)
C13	0.0171 (14)	0.0155 (13)	0.0198 (15)	0.0007 (11)	−0.0061 (11)	0.0024 (11)
C14	0.0142 (13)	0.0105 (12)	0.0146 (13)	−0.0019 (10)	−0.0005 (10)	−0.0007 (10)
C15	0.0137 (13)	0.0110 (12)	0.0127 (13)	−0.0007 (9)	0.0004 (10)	−0.0019 (10)
C16	0.0203 (15)	0.0120 (13)	0.0236 (16)	−0.0054 (11)	−0.0061 (12)	0.0051 (11)
C17	0.0133 (13)	0.0130 (12)	0.0152 (14)	−0.0033 (10)	0.0011 (10)	−0.0006 (10)
C18	0.0128 (13)	0.0163 (13)	0.0185 (14)	−0.0041 (10)	0.0019 (10)	−0.0028 (11)
C19	0.0153 (14)	0.0182 (14)	0.0290 (17)	−0.0044 (11)	0.0005 (12)	0.0027 (12)
C20	0.0238 (16)	0.0179 (14)	0.0315 (18)	−0.0091 (12)	0.0042 (13)	0.0042 (13)
C21	0.0179 (15)	0.0249 (16)	0.0291 (18)	−0.0110 (12)	0.0078 (12)	−0.0063 (13)
C22	0.0117 (14)	0.0304 (17)	0.0261 (17)	−0.0046 (12)	−0.0009 (11)	−0.0051 (13)
C23	0.0151 (14)	0.0179 (14)	0.0214 (15)	−0.0042 (11)	−0.0001 (11)	−0.0016 (11)
C24	0.0273 (17)	0.0250 (16)	0.0246 (17)	−0.0059 (13)	−0.0059 (13)	−0.0078 (13)
C25	0.0276 (18)	0.0260 (17)	0.0298 (19)	−0.0058 (13)	0.0024 (14)	−0.0051 (14)
C26	0.0294 (19)	0.0232 (16)	0.042 (2)	−0.0022 (14)	0.0068 (16)	−0.0011 (15)
Cl1	0.0215 (3)	0.0153 (3)	0.0201 (4)	0.0007 (3)	0.0019 (3)	0.0010 (3)
Cl2	0.0219 (4)	0.0179 (3)	0.0246 (4)	−0.0033 (3)	−0.0068 (3)	−0.0004 (3)
Cl3	0.0184 (3)	0.0265 (4)	0.0237 (4)	−0.0050 (3)	0.0007 (3)	−0.0035 (3)
Dy1	0.01102 (6)	0.00864 (6)	0.01337 (7)	−0.00194 (4)	−0.00055 (4)	0.00032 (4)
N1	0.0149 (11)	0.0099 (10)	0.0194 (13)	−0.0026 (8)	−0.0030 (9)	0.0014 (9)
N2	0.0167 (12)	0.0105 (10)	0.0154 (12)	−0.0017 (9)	−0.0031 (9)	−0.0002 (9)
N3	0.0127 (11)	0.0108 (10)	0.0146 (12)	−0.0020 (8)	−0.0019 (9)	−0.0013 (9)
N4	0.0127 (11)	0.0105 (10)	0.0167 (12)	−0.0028 (8)	−0.0008 (9)	0.0009 (9)
N5	0.0152 (12)	0.0108 (10)	0.0200 (13)	−0.0039 (9)	−0.0041 (9)	0.0027 (9)
O1	0.0145 (10)	0.0109 (9)	0.0213 (11)	−0.0031 (7)	−0.0029 (8)	−0.0019 (8)
O2	0.0146 (10)	0.0142 (9)	0.0259 (12)	−0.0034 (8)	−0.0037 (8)	0.0040 (8)
O3	0.0172 (11)	0.0143 (10)	0.0192 (11)	−0.0022 (8)	−0.0036 (8)	−0.0035 (8)
O4	0.0186 (12)	0.0211 (11)	0.0181 (12)	−0.0082 (9)	0.0020 (9)	0.0000 (9)
O5	0.0241 (12)	0.0130 (10)	0.0181 (11)	−0.0004 (8)	0.0071 (9)	0.0016 (9)
O6	0.0306 (13)	0.0151 (11)	0.0179 (12)	−0.0001 (10)	0.0056 (9)	0.0031 (10)
O7	0.0355 (14)	0.0161 (11)	0.0316 (15)	−0.0042 (10)	0.0083 (11)	0.0005 (10)
O8	0.0306 (13)	0.0260 (13)	0.0272 (14)	−0.0070 (10)	0.0048 (10)	0.0000 (11)

### Triaqua[2,6-diacetylpyridine bis(benzoylhydrazone)]methanoldysprosium(III) trichloride methanol disolvate (DyDAPBH2) Geometric parameters (Å, º)

**Table:**

C1—O1	1.239 (3)	C20—C21	1.384 (5)
C1—N1	1.352 (4)	C20—H20	0.9500
C1—C2	1.481 (4)	C21—C22	1.380 (5)
C2—C7	1.388 (4)	C21—H21	0.9500
C2—C3	1.391 (4)	C22—C23	1.393 (4)
C3—C4	1.386 (4)	C22—H22	0.9500
C3—H3	0.9500	C23—H23	0.9500
C4—C5	1.386 (4)	C24—O3	1.444 (4)
C4—H4	0.9500	C24—H24A	0.9800
C5—C6	1.369 (5)	C24—H24B	0.9800
C5—H5	0.9500	C24—H24C	0.9800
C6—C7	1.387 (4)	C25—O7	1.414 (4)
C6—H6	0.9500	C25—H25A	0.9800
C7—H7	0.9500	C25—H25B	0.9800
C8—N2	1.289 (4)	C25—H25C	0.9800
C8—C10	1.478 (4)	C26—O8	1.427 (4)
C8—C9	1.497 (4)	C26—H26A	0.9800
C9—H9A	0.9800	C26—H26B	0.9800
C9—H9B	0.9800	C26—H26C	0.9800
C9—H9C	0.9800	Dy1—O6	2.313 (2)
C10—N3	1.355 (3)	Dy1—O5	2.354 (2)
C10—C11	1.392 (4)	Dy1—O1	2.358 (2)
C11—C12	1.384 (4)	Dy1—O2	2.3961 (19)
C11—H11	0.9500	Dy1—O4	2.420 (2)
C12—C13	1.386 (4)	Dy1—O3	2.472 (2)
C12—H12	0.9500	Dy1—N4	2.555 (2)
C13—C14	1.393 (4)	Dy1—N2	2.577 (2)
C13—H13	0.9500	Dy1—N3	2.584 (2)
C14—N3	1.348 (4)	N1—N2	1.386 (3)
C14—C15	1.484 (4)	N1—H1A	0.8800
C15—N4	1.287 (4)	N4—N5	1.376 (3)
C15—C16	1.488 (4)	N5—H5A	0.8800
C16—H16A	0.9800	O3—H3S	0.792 (19)
C16—H16B	0.9800	O4—H4SA	0.77 (4)
C16—H16C	0.9800	O4—H4SB	0.73 (4)
C17—O2	1.246 (3)	O5—H5SA	0.81 (5)
C17—N5	1.347 (4)	O5—H5SB	0.73 (5)
C17—C18	1.481 (4)	O6—H6SA	0.77 (4)
C18—C23	1.392 (4)	O6—H6SB	0.66 (4)
C18—C19	1.398 (4)	O7—H7S	0.77 (4)
C19—C20	1.390 (4)	O8—H8S	0.77 (5)
C19—H19	0.9500		
			
O1—C1—N1	120.6 (3)	H24B—C24—H24C	109.5
O1—C1—C2	120.9 (3)	O7—C25—H25A	109.5
N1—C1—C2	118.4 (2)	O7—C25—H25B	109.5
C7—C2—C3	119.5 (3)	H25A—C25—H25B	109.5
C7—C2—C1	122.8 (3)	O7—C25—H25C	109.5
C3—C2—C1	117.5 (2)	H25A—C25—H25C	109.5
C4—C3—C2	120.0 (3)	H25B—C25—H25C	109.5
C4—C3—H3	120.0	O8—C26—H26A	109.5
C2—C3—H3	120.0	O8—C26—H26B	109.5
C5—C4—C3	119.7 (3)	H26A—C26—H26B	109.5
C5—C4—H4	120.1	O8—C26—H26C	109.5
C3—C4—H4	120.1	H26A—C26—H26C	109.5
C6—C5—C4	120.6 (3)	H26B—C26—H26C	109.5
C6—C5—H5	119.7	O6—Dy1—O5	142.37 (9)
C4—C5—H5	119.7	O6—Dy1—O1	75.91 (8)
C5—C6—C7	119.9 (3)	O5—Dy1—O1	139.05 (8)
C5—C6—H6	120.0	O6—Dy1—O2	76.31 (8)
C7—C6—H6	120.0	O5—Dy1—O2	81.41 (8)
C6—C7—C2	120.2 (3)	O1—Dy1—O2	102.05 (7)
C6—C7—H7	119.9	O6—Dy1—O4	143.55 (8)
C2—C7—H7	119.9	O5—Dy1—O4	71.08 (8)
N2—C8—C10	113.6 (2)	O1—Dy1—O4	79.30 (8)
N2—C8—C9	125.8 (3)	O2—Dy1—O4	135.51 (8)
C10—C8—C9	120.5 (2)	O6—Dy1—O3	118.98 (8)
C8—C9—H9A	109.5	O5—Dy1—O3	78.77 (8)
C8—C9—H9B	109.5	O1—Dy1—O3	65.49 (7)
H9A—C9—H9B	109.5	O2—Dy1—O3	68.35 (7)
C8—C9—H9C	109.5	O4—Dy1—O3	72.39 (8)
H9A—C9—H9C	109.5	O6—Dy1—N4	74.67 (8)
H9B—C9—H9C	109.5	O5—Dy1—N4	68.20 (8)
N3—C10—C11	122.3 (3)	O1—Dy1—N4	149.36 (7)
N3—C10—C8	116.2 (2)	O2—Dy1—N4	62.61 (7)
C11—C10—C8	121.3 (2)	O4—Dy1—N4	130.72 (8)
C12—C11—C10	119.0 (3)	O3—Dy1—N4	123.55 (7)
C12—C11—H11	120.5	O6—Dy1—N2	76.91 (8)
C10—C11—H11	120.5	O5—Dy1—N2	126.14 (8)
C11—C12—C13	119.0 (3)	O1—Dy1—N2	62.53 (7)
C11—C12—H12	120.5	O2—Dy1—N2	151.76 (8)
C13—C12—H12	120.5	O4—Dy1—N2	68.03 (8)
C12—C13—C14	119.0 (3)	O3—Dy1—N2	118.59 (7)
C12—C13—H13	120.5	N4—Dy1—N2	117.86 (7)
C14—C13—H13	120.5	O6—Dy1—N3	80.29 (8)
N3—C14—C13	122.5 (2)	O5—Dy1—N3	86.94 (8)
N3—C14—C15	116.2 (2)	O1—Dy1—N3	121.65 (7)
C13—C14—C15	121.3 (3)	O2—Dy1—N3	122.71 (7)
N4—C15—C14	114.3 (2)	O4—Dy1—N3	90.64 (8)
N4—C15—C16	124.5 (2)	O3—Dy1—N3	160.57 (7)
C14—C15—C16	121.1 (2)	N4—Dy1—N3	60.99 (7)
C15—C16—H16A	109.5	N2—Dy1—N3	60.48 (7)
C15—C16—H16B	109.5	C1—N1—N2	114.6 (2)
H16A—C16—H16B	109.5	C1—N1—H1A	122.7
C15—C16—H16C	109.5	N2—N1—H1A	122.7
H16A—C16—H16C	109.5	C8—N2—N1	119.1 (2)
H16B—C16—H16C	109.5	C8—N2—Dy1	122.84 (18)
O2—C17—N5	119.6 (2)	N1—N2—Dy1	114.88 (16)
O2—C17—C18	121.6 (3)	C14—N3—C10	117.9 (2)
N5—C17—C18	118.8 (2)	C14—N3—Dy1	121.69 (17)
C23—C18—C19	120.2 (3)	C10—N3—Dy1	120.38 (18)
C23—C18—C17	117.4 (3)	C15—N4—N5	117.9 (2)
C19—C18—C17	122.4 (3)	C15—N4—Dy1	126.39 (18)
C20—C19—C18	120.1 (3)	N5—N4—Dy1	115.65 (17)
C20—C19—H19	120.0	C17—N5—N4	116.2 (2)
C18—C19—H19	120.0	C17—N5—H5A	121.9
C21—C20—C19	119.4 (3)	N4—N5—H5A	121.9
C21—C20—H20	120.3	C1—O1—Dy1	125.95 (18)
C19—C20—H20	120.3	C17—O2—Dy1	125.06 (18)
C22—C21—C20	120.7 (3)	C24—O3—Dy1	128.33 (19)
C22—C21—H21	119.7	C24—O3—H3S	107 (3)
C20—C21—H21	119.7	Dy1—O3—H3S	115 (3)
C21—C22—C23	120.6 (3)	Dy1—O4—H4SA	122 (3)
C21—C22—H22	119.7	Dy1—O4—H4SB	122 (3)
C23—C22—H22	119.7	H4SA—O4—H4SB	104 (4)
C18—C23—C22	119.0 (3)	Dy1—O5—H5SA	115 (3)
C18—C23—H23	120.5	Dy1—O5—H5SB	125 (4)
C22—C23—H23	120.5	H5SA—O5—H5SB	116 (5)
O3—C24—H24A	109.5	Dy1—O6—H6SA	118 (3)
O3—C24—H24B	109.5	Dy1—O6—H6SB	126 (3)
H24A—C24—H24B	109.5	H6SA—O6—H6SB	116 (4)
O3—C24—H24C	109.5	C25—O7—H7S	114 (3)
H24A—C24—H24C	109.5	C26—O8—H8S	107 (4)

### Triaqua[2,6-diacetylpyridine bis(benzoylhydrazone)]methanoldysprosium(III) trichloride methanol disolvate (DyDAPBH2) Hydrogen-bond geometry (Å, º)

**Table:**

*D*—H···*A*	*D*—H	H···*A*	*D*···*A*	*D*—H···*A*
O4—H4*SA*···Cl3	0.77 (4)	2.45 (4)	3.211 (3)	170 (4)
N1—H1*A*···Cl2^i^	0.88	2.53	3.298 (2)	147
O6—H6*SA*···Cl1^i^	0.77 (4)	2.27 (4)	3.030 (3)	168 (4)
O3—H3*S*···Cl3^ii^	0.79 (3)	2.60 (3)	3.329 (2)	156 (3)
O4—H4*SB*···Cl3^iii^	0.73 (5)	2.42 (4)	3.133 (3)	165 (4)
O6—H6*SB*···Cl1^iv^	0.67 (3)	2.39 (3)	3.058 (3)	179 (5)
O7—H7*S*···Cl2^v^	0.78 (4)	2.29 (4)	3.049 (3)	165 (4)
O8—H8*S*···Cl3^vi^	0.77 (5)	2.34 (5)	3.104 (3)	174 (6)
O5—H5*SA*···O8^vi^	0.81 (4)	1.96 (4)	2.702 (4)	154 (4)
O5—H5*SB*···O7^v^	0.72 (5)	1.95 (5)	2.659 (3)	167 (6)
C7—H7···Cl2^i^	0.95	2.75	3.494 (3)	136
C11—H11···Cl1^vii^	0.95	2.80	3.730 (3)	167
C12—H12···Cl1^viii^	0.95	2.79	3.734 (3)	172
C16—H16*B*···Cl2	0.98	2.66	3.620 (3)	166
C16—H16*C*···Cl2^viii^	0.98	2.77	3.618 (3)	145
C19—H19···Cl2	0.95	2.74	3.522 (3)	140
C26—H26*A*···Cl3^ix^	0.98	2.79	3.761 (4)	171
C4—H4···O8^x^	0.95	2.60	3.406 (4)	143

Symmetry codes: (i) *x*, *y*−1, *z*; (ii) −*x*+1, −*y*, −*z*+1; (iii) −*x*, −*y*, −*z*+1; (iv) −*x*+1, −*y*+1, −*z*; (v) −*x*+1, −*y*+1, −*z*+1; (vi) −*x*, −*y*+1, −*z*+1; (vii) *x*−1, *y*−1, *z*; (viii) −*x*, −*y*+1, −*z*; (ix) *x*, *y*+1, *z*; (x) *x*+1, *y*−1, *z*.
